# Dissecting seipin function: the localized accumulation of phosphatidic acid at ER/LD junctions in the absence of seipin is suppressed by Sei1p^ΔNterm^ only in combination with Ldb16p

**DOI:** 10.1186/s12860-015-0075-3

**Published:** 2015-12-04

**Authors:** Sungwon Han, Derk D. Binns, Yu-Fang Chang, Joel M. Goodman

**Affiliations:** Department of Pharmacology, University of Texas Southwestern Medical School, Dallas, TX 75390-9041 USA; Department of Molecular Biology and Genetics, Cornell University, Ithaca, NY 14853-2703 USA

**Keywords:** Seipin, Lipid droplets, Phosphatidic acid, FLD1, SEI1, LDB16

## Abstract

**Background:**

Seipin is required for the correct assembly of cytoplasmic lipid droplets. In the absence of the yeast seipin homolog Sei1p (formerly Fld1p), droplets are slow to bud from the endoplasmic reticulum, lack the normal component of proteins on their surface, are highly heterogeneous in size and shape, often bud into the nucleus, and promote local proliferation of the endoplasmic reticulum in which they become tangled. But the mechanism by which seipin catalyzes lipid droplet formation is still uncertain.

**Results:**

Seipin prevents a localized accumulation of phosphatidic acid (PA puncta) at ER-droplet junctions. PA puncta were detected with three different probes: Opi1p, Spo20p(51–91) and Pah1p. A system of droplet induction was used to show that PA puncta were not present until droplets were formed; the puncta appeared regardless of whether droplets consisted of triacylglycerol or steryl ester. Deletion strains were used to demonstrate that a single phosphatidic acid-producing enzyme is not responsible for the generation of the puncta, and the puncta remain resistant to overexpression of enzymes that metabolize phosphatidic acid, suggesting that this lipid is trapped in a latent compartment. Suppression of PA puncta requires the first 14 amino acids of Sei1p (Nterm), a domain that is also important for initiation of droplet assembly. Consistent with recent evidence that Ldb16p and Sei1p form a functional unit, the PA puncta phenotype in the *ldb16Δ sei1Δ* strain was rescued by human seipin. Moreover, PA puncta in the *sei1Δ* strain expressing Sei1p^ΔNterm^ was suppressed by overexpression of Ldb16p, suggesting a functional interaction of Nterm with this protein. Overexpression of both Sei1p and Ldb16p, but not Sei1p alone, is sufficient to cause a large increase in droplet number. However, Ldb16p alone increases triacylglycerol accumulation in the *ldb16Δ sei1Δ* background.

**Conclusion:**

We hypothesize that seipin prevents formation of membranes with extreme curvature at endoplasmic reticulum/droplet junctions that would attract phosphatidic acid. While Ldb16p alone can affect triacylglycerol accumulation, proper droplet formation requires the collaboration of Sei1p and Ldb16.

**Electronic supplementary material:**

The online version of this article (doi:10.1186/s12860-015-0075-3) contains supplementary material, which is available to authorized users.

## Background

Cytoplasmic lipid droplets, found in most eukaryotic and some prokaryotic cells, store energy in the form of neutral lipids, particularly triacylglycerols (TAG) and steryl esters (SE), which are enwrapped by a single phospholipid monolayer and associated proteins [1]. They emanate from the endoplasmic reticulum (ER) and can remain attached there [2]; they also generate contacts with other organelles, presumably for the purpose of lipid exchange [3, 4].

The synthesis of droplets is becoming better understood. The general view of early steps is that collections of neutral lipids, as they accumulate between the ER leaflets after synthesis, eventually will assume a spherical shape, remaining covered with ER membrane as they bud outward [5, 6]. However, proteins may control this reaction [7]. For example, Fit2 is found in the ER, binds to neutral lipids in isolation, and clearly affects droplet formation in some systems [8, 9]. Our laboratory has been focusing on seipin, a protein first identified in severe congenital generalized lipodystrophy, in which adipose tissue fails to develop [10].

Seipin was also identified in yeast screens for morphologically aberrant lipid droplets [11, 12]. The absence of seipin in yeast leads to both irregularly shaped clusters of tiny droplets enwrapped in the ER, and “supersized” droplets; the two forms depends on levels of cell phospholipids, as the supersized phenotype is suppressed by high levels of the phospholipid precursor, inositol [13]. Droplets in seipin-null cells also frequently bud into the nucleus, a phenomenon rarely seen in wild type cells [14]. Moreover, they lack, or have diminished levels, of a subset of droplet proteins [15, 16].

The absence of seipin also results in slow formation of droplets under conditions where the amount of total cellular neutral lipid is identical. When droplets are produced in this system, they are often actually clusters of microdroplets with ER fragments attached, as if a product of bilayer membrane stability [14]. Overall, these studies suggest that seipin is important in controlling the initiation of droplets as well as affecting the size, topology and protein composition of the droplets. How it performs these functions on a molecular basis, however, is unclear.

Seipin spans the ER membrane twice [17]. Yeast seipin (Sei1p, formally Fld1p) has two short ends facing the cytosolic side. We recently reported that the 14 amino acids comprising the amino-terminal domain (“Nterm”) was critical for a normal rate of initiation of droplet formation but not for droplet morphology or vectorial budding (towards the cytoplasm vs. nucleus). However, while droplets are spherical in the Sei1^ΔNterm^ mutant (compared to irregularly shaped in *sei1Δ*), they are larger, consistent with poor initiation yielding fewer droplets. Sei1p binds to Ldb16p, and the double deletion is complemented by human seipin [15], suggesting that Sei1p/Ldb16p is the seipin functional unit.

We now report a novel phenotype of seipin-null cells: a punctate pattern of phosphatidic acid (PA) in the ER adjacent or close to droplets. (Note: Independently, another report of PA puncta in seipin-null cells appeared shortly before submission of this work [18].) These PA puncta are not present in cells lacking droplets, and they appear as droplets are formed. Our data are consistent with the puncta representing aberrant membrane curvature serving as a sink for wedge-shaped lipids, which is normally avoided or covered by seipin during normal droplet assembly and maintenance. We further report that Ldb16p may play a role independent of Sei1p in accumulation of triacylglycerols.

## Results

### PA accumulates in puncta in seipin-KO cells

**Fig 1:**
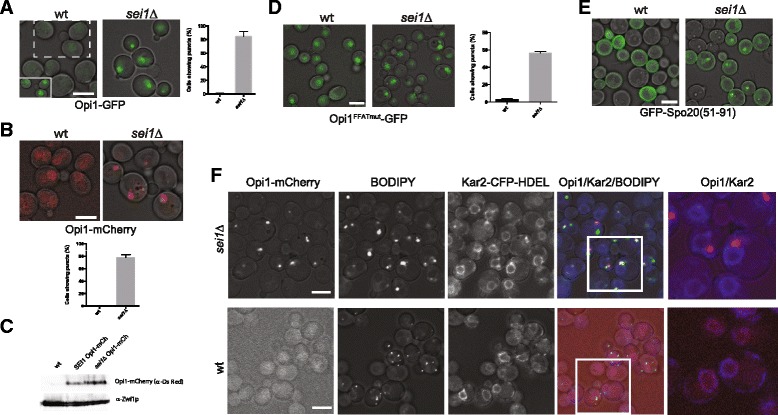
Phosphatidic acid (PA) is concentrated in nuclear ER and lipid droplets in *sei1*Δ. **a** Opi1-GFP localization in wild type and in *sei1Δ* cells. *Left*, merged fluorescent and brightfield images are shown; equivalent intensity settings. Inset: contrast is enhanced in the area enclosed in dashed rectangle to more clearly show nuclear staining of Opi1-GFP in wild type cells. *Right*, percentage of cells with punctate staining. Average ± SE from three experiments, > 90 cells counted in each. **b** As in (A) but with Opi1-mCherry in W303 strain. **c** Immunoblot of Opi1-mCherry in wild type and *sei1Δ* cells, representative of three experiments. Signals were quantified using an Infrared detection system (Odyssey LiCoR Scanner), which showed that the Opi1-mCh signal was higher than that of wt (1.8 ± 0.2 fold). The cytoplasmic marker Zwf1p is used as a control. **d** Mutation of the FFAT motif in the chromosomal copy of Opi1-GFP reduces but does not eliminate the PA puncta. Left, GFP fluorescence; right, quantification of the percentage of cells with puncta. **e** Fluorescence microscopy of wild type and *sei1Δ* cells (W303 background) expressing GFP-Spo20(51–91). As in (A) but with GFP-Spo20(51–91). **f** Cells co-expressing Opi1-mCherry and Kar2-CFP-HDEL (ER marker) were stained with BODIPY 493/503. In the first merged images (fourth column), the BODIPY channel is green, Opi1-mCherry red, and Kar2-CFP-HDEL blue. The second merged images (fifth column) are magnifications of the preceding white boxes, illustrating Kar2 (ER, blue) and Opi1 (red). Scale bars, 5 μm., and all micrographs correspond to single optical sections. For quantification of cells with PA-puncta, at least 90 cells were counted in each of at least three independent experiments

A previous report demonstrated a 20 % increase in phosphatidic acid (PA) in enriched ER membranes in seipin-KO (knockout) yeast cells [13]. To confirm this biochemical finding and determine whether the PA was associated with an ER subdomain, we tagged Opi1p, a phosphatidic acid sensor [19], in the genome with GFP in cells containing or lacking *SEI1* (yeast seipin, formerly *FLD1*). Under inositol-replete nutrient conditions, Opi1p is nuclear and represses the synthesis of several genes in phospholipid biosynthesis. However, during inositol starvation, Opi1p traffics to the ER where it binds to PA, which accumulates there [19]. Wild type cells displayed weak Opi1p-GFP nuclear fluorescence when cultured in defined SCD medium (Fig. 1a). However, bright Opi1p-GFP puncta were observed in 85 % of *sei1Δ* cells under the same conditions, suggesting a local accumulation of PA. An identical phenotype was seen with Opi1p-mCherry, indicating the pattern was not related to a specific fluorescent tag (Fig. 1b). The bright Opi1p puncta in *sei1Δ* compared with the dim nuclear staining probably represents a combination of high local concentration and increase in Opi1p protein – indeed, cellular Opi1p-mCherry is increased about 80 % of the *sei1Δ* strain, for unknown reasons (Fig. 1c).

While membrane PA is a determinant of Opi1p binding to ER membranes, other factors come into play: Opi1p also binds to Scs2p in the ER through its FFAT sequence [20], and the Opi1p-Scs2p complex can bind to the Yet1p-Yet3p complex [21]. Furthermore, Opi1p favors PA containing esterified C16 vs. C18 fatty acids [22]. To rule out Scs2p binding, in the Opi1p puncta, we tested the effect of a Opi1p FFAT loss-of-function mutation (EEFD200-203ALLA [19]) on the punctate pattern in *sei1Δ* and still observed puncta in most cells (Fig. 1d). We found no difference in Scs2p-GFP localization in wild type and *sei1Δ* strains except a slight increase in punctate Scs2p-GFP in 10 % of stationary-phase cells cells (Additional file 1: Figure S1); this cannot account for the prominent Opi1p puncta. To confirm that the Opi1p puncta represent concentrated PA, we used another PA sensor, a fragment of Spo20p (aa 51–91) conjugated to GFP [23]. In wild type cells the Spo20p fragment bound to membranes, especially at the cell periphery. However, in the *sei1Δ* strain much of Spo20p(51–91)-GFP was also localized to puncta (Fig. 1e), confirming that PA is highly localized in the absence of seipin.

Sei1p localizes to ER at the junction of lipid droplets and is important for initiation of droplet formation and controlling droplet morphology, in which droplets are normally uniform in shape and size and are dispersed along the ER. We hypothesize that the concentrated PA in the absence of seipin leads to aberrant morphology observed by three groups: clustered droplets in ER tangles and a large range of sizes [11, 12, 16]. If this is the case the PA puncta may colocalize with the ER-droplet aggregates. This was clearly the case, as the puncta colocalized or were adjacent to both droplets, visualized with BODIPY 493/503, and were on the ER, detected with Kar2-CFP-HDEL (Fig. 1f) or with Sec63-CFP (not shown). These data suggested that the PA puncta and aberrant droplets in the absence of seipin were related phenomena.

**Fig. 2:**
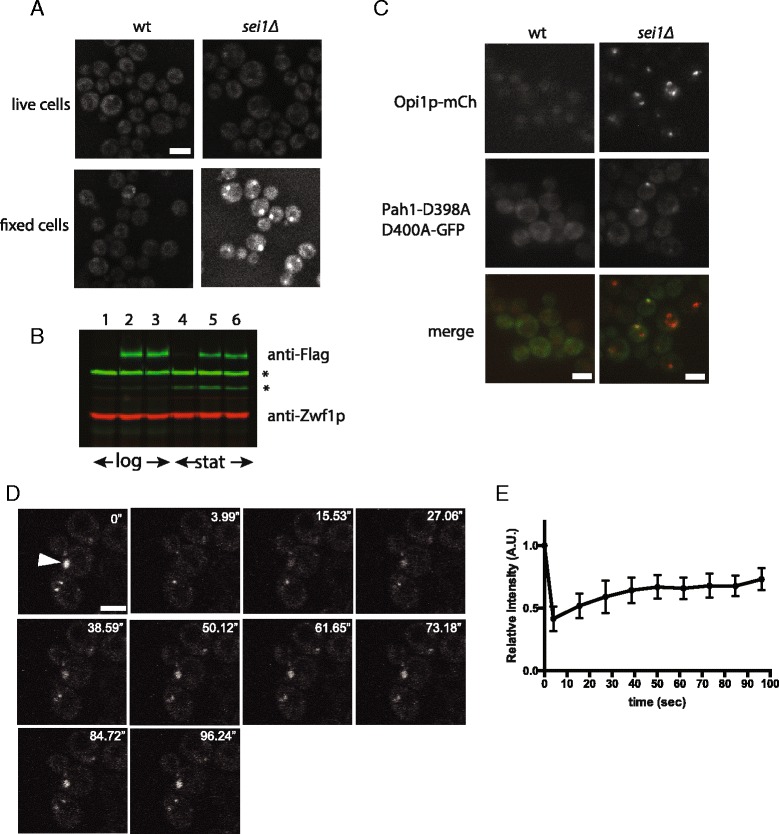
Pah1p also forms puncta in *sei1*Δ cells. **a** Chromosomally tagged Pah1p with GFP (Pah1-GFP) in wt and *sei1Δ* cells (BY4741 background). Puncta were apparent in *sei1Δ* when cells were fixed with 2 % formaldehyde solution but not in living cells. **b** Pah1p-FLAG was expressed similarly in wt and *sei1Δ* cells (W303 background). Lanes 1–3 mid-log cells; lanes 4–6 stationary phase cells. Lanes 1,4 wt; lanes 2,5 FLAG knock-in behind *PAH1* ORF in wt; lanes 3,6 FLAG knock-in behind *PAH1* ORF in *sei1Δ* cells. Whole cell lysates were immunoblotted with anti-FLAG and anti-Zwf1p and imaged by infrared fluorescence detection method (Odyssey™ Western blots). Asterisks, non-specific bands. **c** Catalytically-dead Pah1p forms dots that colocalize with Opi1p-puncta. Wild-type or *sei1Δ* cells, both containing the Opi1-mCherry allele were transformed with plasmid encoding Pah1p(D398A D400A)-GFP. Living cells were imaged, illustrating colocalized markers (Opi1p-mCh and Pah1(D398A D400A)-GFP colored red and green, respectively, in the merged images). **d** Dots of Pah1p(D398A D400A)-GFP in *sei1Δ* Opi1-mCherry recover slowly after photobleaching. After bleaching the indicated punctum with laser, recovery is slow, as shown by time-lapse (10 s interval). **e** Quantification of fluorescence recovery. The intensities were normalized to the initial intensity of the each puncta before bleaching. Data represent 5 bleaching experiments, mean ± SEM

We also detected puncta with Pah1p, the phosphatidic acid phosphatase that is the ortholog to mammalian lipin [24]. Most of Pah1p is cytosolic. However, more of it can be driven to ER membranes by overexpression of the DAG kinase, Dgk1p, presumably by an increase in phosphatidic acid, the product of Dgk1p [25]. Pah1p puncta also has been demonstrated very recently by new lipid droplets [26]. While membrane localization of Pah1p-GFP in living *sei1*Δ cells was not apparent in our hands, strong puncta occur in cells fixed with 2 % (v/v) formaldehyde (Fig. 2a); the strong puncta was not a result of higher expression of Pah1p in *sei1Δ,* as it was expressed (with FLAG tag) similarly in both strains (Fig. 2b). The fixative may have trapped the enzyme on the membrane, perhaps by not allowing it to metabolize the PA to which it is bound. To determine whether these spots represent PA puncta, we used a catalytically dead mutant, Pah1-D398A/D400A-GFP [25]. This protein clearly localizes to puncta in living cells, and the spots colocalize to Opi1p-mCherry (Fig. 2c). Thus, Pah1-D398A/D400A-GFP appears to be another PA sensor, albeit not as sensitive as Opi1p. A fluorescence bleaching-recovery experiment indicated that there was only slow (plateau after ~ 50 s) and incomplete replacement of bleached Pah1-D398A/D400A-GFP, suggesting that the PA pool in the puncta is relatively stable (Fig. 2d and e).

### The PA puncta appear during lipid droplet formation

**Fig. 3:**
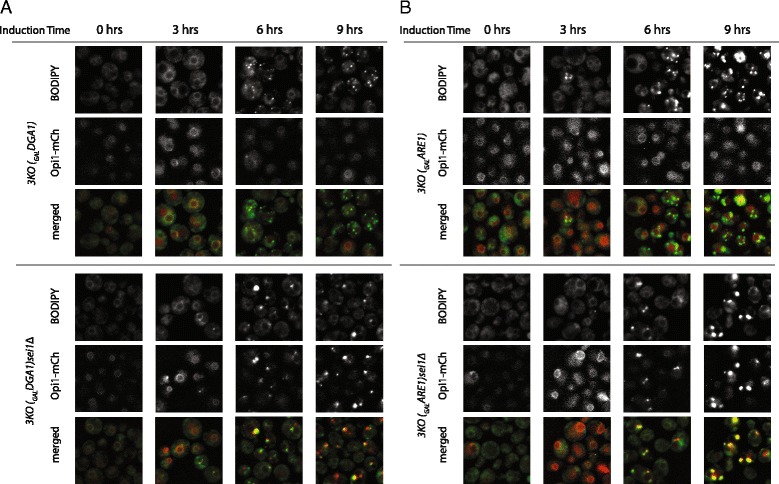
PA puncta appear with TAG and SE droplets in *sei1Δ* strains. Yeast strains chromosomally expressing Opi1-mCherry, with or without a *SEI1* allele, and which only produce neutral lipid upon addition of galactose ((**a**) 3KO(_GAL_DGA1) produces TAG, while (**b**) 3KO(_GAL_ARE1) produces steryl ester, SE [14]), were subjected to galactose at 0 time and monitored every three hours. PA puncta (marked by Opi1-mCherry) became apparent only in *sei1Δ* cells and only upon formation of lipid droplets, detected by BODIPY. PA puncta were present in both TAG and SE droplets

We then asked whether the appearance of PA puncta, which were generally adjacent to droplets on the ER in *sei1Δ* cells, required droplets. This was tested in strains that are deleted in all but one enzyme that synthesizes neutral lipids for droplets, and in which the expression of the remaining enzyme is under control of the galactose promoter [14, 27]. Two such strains were utilized in which TAG or SE droplets form subsequent to induction of *DGA1* or *ARE1*, respectively, in galactose-containing medium. The endogenous copy of *OPI1* was tagged in these strains with mCherry to allow detection of PA puncta. No PA puncta were observed in cells containing *SEI1* when *DGA1* was induced, as expected (Fig. 3a). Puncta were also absent in cells also deleted in *SEI1* when growing in glucose. However, upon induction of *DGA1*, PA puncta were observed as droplets appeared (at 3 h). At this time point there were many droplets without clear puncta, although each punctum overlapped or was adjacent to droplets, suggesting that droplet formation in the absence of seipin may precede and even cause development of puncta.

We considered that the PA puncta in *sei1Δ* cells were related to TAG synthesis by Dga1p, as PA is a precursor of TAG. This was not the case. If droplet formation was driven instead by the steryl acetyltransferase Are1p, resulting in steryl ester formation and very little TAG, we saw the identical behavior: bright PA puncta upon formation of new droplets (Fig. 3b). PA puncta, therefore, form in seipin-null cells as droplets form regardless of the neutral lipid or synthetic enzyme producing them.

### No single enzyme in PA metabolism is responsible for the PA puncta

**Fig. 4:**
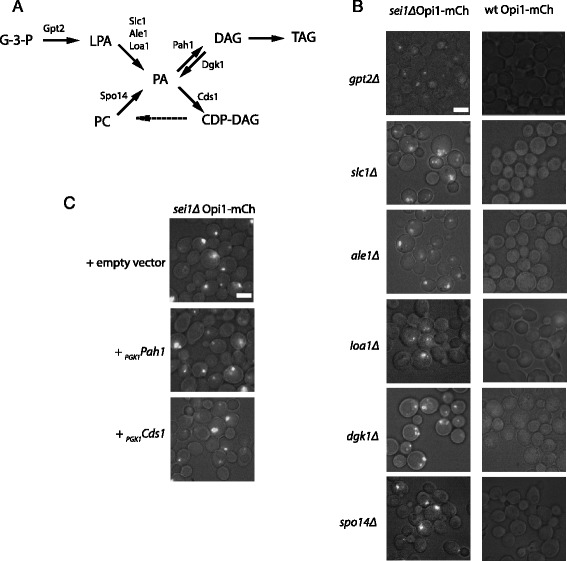
Manipulation of single metabolic enzymes do not eliminate PA puncta. **a** Diagram of pathways leading to PA production and use. **b**, **c** Genetic manipulations were performed in wt and *sei1Δ* strains, each expressing mCherry behind OPI1 ORF in the chromosome. **b** The indicated (at left) enzymes producing PA were knocked out. All resulting strains still produced PA puncta. **c** The two enzymes that metabolize PA, Pah1p and Cds1p, were overexpressed on plasmids using the *PGK1* promoter. Neither resulting strain attenuated the PA puncta. Scale bars, 5 μm

We attempted to identify an enzyme related to PA that was responsible for either the synthesis of the PA in the puncta or their absence in the presence of Sei1p (Fig. 4a). PA can be generated from glycerol-6-phosphate (G-3-P) by acyltransferases in two steps: first to lysophosphatidic acid (LPA) by Gpt2p, then to PA by Loa1p, Ale1p, or Slc1p. PA can also be generated from phosphatidyl choline by the phospholipase D, Spo14p, or from diacylglycerol (DAG) by the DAG kinase Dgk1p. We deleted each of these enzymes in strains containing mCherry-tagged Opi1p with or without *SEI1*. As expected, there were no PA puncta in the strains containing *SEI1* (Fig. 4b). Conversely, all strains in the *sei1Δ* contained puncta, ruling out the possibility that one PA synthetic enzyme was uniquely responsible for them.

We also attempted to eliminate the puncta by overexpressing enzymes that metabolized PA. However, high expression of Pah1p (which converts PA to DAG) or Cds1p (which converts PA to CDP-DAG) failed to attenuate the Opi1-mCherry puncta in the *sei1Δ* strain (Fig. 4c), suggesting that the puncta are not a result of suppression of these enzymes in the absence of seipin, and also that these enzymes do not have good access to the puncta. To attempt to target these two enzymes to the PA puncta, we generated fusion proteins between Cds1p or Pah1p and Sei1^ΔNterm^ (Sei1p missing the first 14 amino acids, which partially targets to ER-droplet junctions [14] but does not suppress PA puncta; see below). The Cds1p-Sei1^ΔNterm^ fusion had no effect on the PA puncta (data not shown), while the cells expressing Pah1p- Sei1^ΔNterm^ were largely dead and were not further analyzed.

Formation of supersized droplets that accumulate in *sei1Δ* cells can be suppressed by addition of 75 μM inositol to the medium, which results in an increase in phospholipid synthesis [13]. We found that suppression of supersized droplets by inositol addition did not inhibit the accumulation of PA puncta in *sei1Δ* cells (Additional file 1: Figure S2).

In summary, none of our genetic manipulations of single genes to lower PA levels eliminated the puncta, suggesting that PA from multiple sources was trapped there, and that it was not easily accessible as substrate to enzymes for further metabolism.

### PA puncta disappear in the presence of human seipin or a combination of Sei1p^ΔNterm^ and Ldb16p

**Fig 5:**
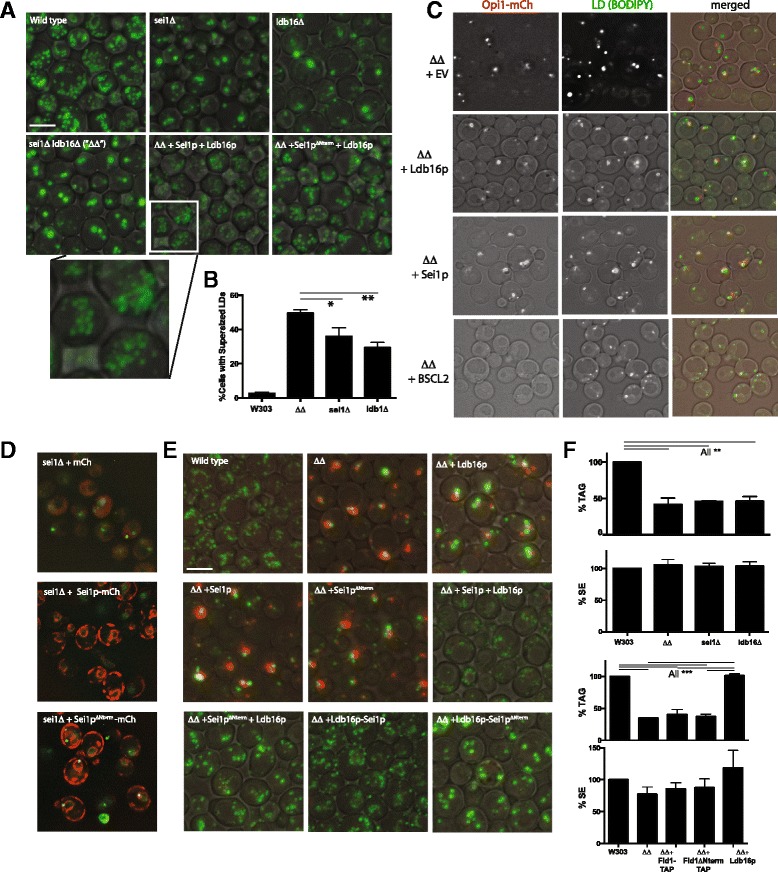
Sei1^ΔNterm^ suppresses puncta but only in the presence of overexpressed Ldb16p. **a** BODIPY staining of droplets in the indicated yeast strains. The multiple and less distinct droplets in the ΔΔ + Sei1p + Ldb16p strain is shown in greater magnification at the bottom. **b** Quantification of cells with supersized (>1 μm diameter) droplets in the indicated strains. Three fields of >240 cells/field were counted from two independent experiments. Error bars signify SEM. Lines above the bars indicate statistical difference between the indicated pairs, based on the Turkey range test in conjunction with ANOVA. *, *p* < 0.05; **, *p* <0.01. **c** Human seipin (BSCL2) suppresses PA-puncta in the ΔΔ strain. **d** PA-puncta in *sei1*Δ Opi1-GFP (BY4742) are not suppressed by Sei1^ΔNterm^-mCherry. An overexpressed mCherry fusion was used to illustrate the correct ER targeting. **e** Puncta are absent with the combination of Sei1^ΔNterm^ and Ldb16. Green, BODIPY493/503; red, Opi1-mCherry. **f** Overexpressed Ldb16 in the ΔΔ background increases TAG but not SE levels. TAG and SE in the W303 wild type control strain set at 100 %. Bars and error bars represent mean + range from two independent experiments. Lines above the bars indicate statistical difference between the indicated pairs, based on the Turkey range test in conjunction with ANOVA. **, *p* <0.01; ***, *p* < 0.001. Scale bars, 5 μm

Recently Ldb16p was identified as a binding partner for Sei1p [15]. Knocking out *LDB16* produced a droplet phenotype similar to *sei1Δ*. The double knockout could be rescued by human seipin, suggesting that Sei1p-Ldb16 formed a functional seipin complex [15]. Consistent with this report we also observed similar droplet phenotypes of *sei1Δ* and *ldb16Δ*, namely a combination of droplet clusters (diffuse BODIPY pattern) and supersized droplets (Fig. 5a); we detected no droplet morphological difference between these two strains. Interestingly, in a strain in which both genes were deleted (“ΔΔ”), we observed significantly more cells with supersized droplets (Fig. 5b), suggesting that the frequent droplet clusters observed in the single knockouts may be a gain-of-function phenotype of expression of one of the partners alone. Expression of both proteins in the strain under the strong *PGK1* promoter resulted in a different phenotype: a proliferation of very small droplets (too many to count) dispersed throughout the cytoplasm (Fig. 5a). Yeast Sei1p has been shown to be important for initiation of droplet assembly [14]. This experiment suggests there are no other limiting factors for droplet production other than Sei1p and Ldb16p.

We recently showed that removal of the amino terminal 14 amino acids from seipin (generating Sei1^ΔNterm^) resulted in a partial phenotype compared to the null strain: droplet initiation was slower, resulting in larger droplets, but the droplets appeared otherwise normal [14]. Similarly, overexpression of Sei1^ΔNterm^ with Ldb16p in the double deletion strain resulted in a homogeneous population of droplets larger than found in the full-length Sei1p control (Fig. 5a).

PA puncta were observed in the ΔΔ strain (Fig. 5c). Replacement of either protein alone did not result in their disappearance. However, no puncta were observed upon expression of human seipin (BSCL2). As human seipin can complement yeast seipin null strains (*sei1Δ* and *sei1Δ ldb16Δ*) in promoting droplet formation [15], our data suggest that preventing PA puncta is a conserved role of seipin.

Sei1^ΔNterm^, even when overexpressed, was unable to cause the disappearance of PA puncta in the *sei1* null strain (Fig. 5d), and caused the appearance of PA puncta even when expressed in the wild type strain (not shown); this dominant negative effect was seen before in regards to droplet morphology [14]. Surprisingly, PA puncta were suppressed when Sei1^ΔNterm^ was co-overexpressed with Ldb16, either as independent proteins or as a tethered unit, Ldb16- Sei1^ΔNterm^ (Fig. 5e). Therefore, while the two proteins together produce larger droplets (consistent with the role of the Sei1p amino terminal domain in droplet initiation), this phenotype can be dissected away from the appearance or PA puncta.

In our system, deleting *SEI1*, *LDB16*, or both resulted in a strong decrease in cellular TAG with no effect of SE levels (Fig. 5f and data not shown). Replacement with overexpressed Sei1p or Sei1p^ΔNerm^ in the double deletion mutant did not increase TAG. However, overexpression of Ldb16p alone in the double deletion strain restored TAG to normal levels. This result indicates that Ldb16 can function independently of Sei1p to affect droplet size and TAG accumulation.

## Discussion

In this report we show a strong and localized accumulation of PA in ER membranes of cells that lack seipin. PA accumulation only occurred in cells with droplets and seemed to occur concomitant or shortly after droplet formation. We could find no single enzyme that produces PA to account for the localized puncta, nor an overexpressed enzyme that normally utilizes PA to make the puncta disappear (although we cannot rule out that Opi1p is sequestering the PA from metabolic enzymes). The study of strains with multiple knockouts in PA-related pathways is likely to provide more information. Nevertheless, based on our experiments thus far, we conclude that the puncta likely represents a sink of PA that is unavailable to utilizing enzymes.

**Fig. 6:**
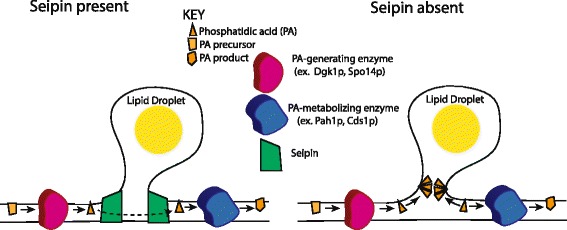
Working model for PA puncta in seipin-deleted cells. ER-droplet interface is shown. Left, normally, PA fluxes from generating to metabolizing enzymes. PA flux through the TAG pathway into droplets is not specifically shown. Curvature at the junction is mild or it protected by seipin. Right, in the absence of seipin, some PA is trapped at the ER-drop interface in regions of high curvature, creating punta seen by fluorescence probes

PA is a wedge-shaped molecule that would be expected to intercalate into curved membranes. Our data are consistent with a model in which the radical curvature that accompanies droplet budding will trap wedge-shaped molecules such as phosphatidic acid. As phosphatidic acid is at the nexus of phospholipid and neutral lipid synthesis, and the ER is home to many enzymes in these pathways, the flux through PA in the ER should be high, allowing for trapping of this metabolic intermediate (Fig. 6, right). Changes in metabolic fluxes may also affect the intensity of the puncta, but our data suggest that such fluxes are not responsible for the puncta themselves.

Seipin may prevent PA trapping by providing the curvature itself, thereby blocking the insertion of PA (Fig. 6, left). Such a role would be consistent with its function in initiating droplet formation [14] as the seipin-induced curvature could facilitate the entry of neutral lipids into a nascent bud. Seipin also could be acting more indirectly: By facilitating droplet formation, it may orchestrate a more gentle process by which spontaneous blebbing of lipid drops is avoided which may be accompanied by more severe bilayer curvature at trapping of PA. Necks the ER-droplet junctions have been observed in which the curvature is gentle [28].

It is interesting that overexpressing both Sei1p and Ldb16p results in many more lipid droplets than wild type (Fig. 5a). Overexpressing Sei1p alone does not result in more droplets, which was concerning if seipin played a role in droplet initiation [12]. The large increase in droplet number upon overexpression of both proteins is consistent with these two subunits being sufficient for droplet formation with no other proteins required in stoichiometric amounts.

In our studies deletion of either *SEI1*, *LBD16* or both resulted in a 50-60 % decrease in TAG accumulation (Fig. 5f). This is contradictory to an earlier report showing an increase in TAG in the absence of SEI1 [11]. Moreover, several reports have shown a tissue-specific decrease in TAG with seipin overexpression [29]. In our hands we find that the decrease in TAG is more severe in defined medium (as used here) compared to rich YPD broth. Although a decrease in TAG in the null strain is consistent with the lipodystrophy phenotype in patients [10], it is difficult to relate effects on adipogenesis in the mammalian system with effects seen here in yeast. Nevertheless, it is interesting that the decrease in TAG in the ΔΔ strain is suppressed in the presence of overexpressed Ldb16p. This protein was shown to be unstable in the absence of Sei1p [15]. Our result indicates that enough protein remains upon overexpression to significantly increase TAG accumulation. Ldb16p may have this effect by directly influencing TAG synthesis (by activating Dga1p or Lro1p) or by increasing the rate of lipolysis or lipophagy. While Ldb16p in the ΔΔ strain suppressed the decrease in TAG, it did not suppress PA puncta formation, indicating that the two phenomena are not linked. Regardless of mechanism, it is the first observation that Ldb16 functions in lipid storage independent of its binding partner, and it is consistent with the observation that a subpopulation of Ldb16p does not colocalize with Sei1p [15].

The functions of seipin can be dissected by the elimination of the first fourteen amino acids of Sei1p: the small clusters of droplets enwrapped in ER that are found in *sei1Δ* null cells are not formed, and frequent droplets in the nucleus are also avoided. Initiation of droplet assembly is still sluggish however, which is consistent with larger and fewer droplets in cells [14]. We tethered Ldb16p to Sei1^ΔNterm^ to determine if the defect in the truncated form may be related to its ability to bind properly to Ldb16p, although previous data showed that the transmembrane and loop regions of Sei1p were important for pull down of the two proteins [15]. Indeed, cells expressing the tethered form lacked PA puncta (Fig. 5). However, droplets were still larger than normal in the strain, suggesting the tethered proteins could not restore a normal rate of droplet initiation. The phenotype was similar if the two proteins were singly expressed. These results suggest that avoiding PA puncta is not sufficient for seipin to generate wild type droplets. It is possible that Ldb16p-Sei1p^ΔNterm^ can deform the membrane sufficiently to avoid trapping PA but not to the extent required for a normal rate of droplet initiation.

In summary, we have shown the presence of PA puncta in seipin null cells and suggest that these are caused by extreme curvature of the membrane, attracting wedge-shaped lipids such as PA. Puncta are not formed until droplets appear, and the puncta are formed regardless of neutral lipid types that comprise the core. Our data using overexpressed Ldb16p shows its ability to suppress the PA puncta produced by Sei1p^ΔNterm^ and that it can regulate TAG levels independent of Sei1p.

## Conclusions

The functional seipin complex in yeast is comprised of Sei1p and Ldb16p. Seipin is shown here to prevent the localized accumulation of phosphatidic acid in the ER adjacent to development of lipid droplets. The accumulation only occurs with droplet formation and occurs for both triacylglycerol (TAG) and steryl ester (SE) droplets. Sei1p lacking its amino terminus is not sufficient to prevent droplet formation, but this defect can be suppressed by overexpressing Ldb16p. Lack of either Sei1p or Ldb16p strongly inhibits the accumulation of TAG but not SE in cells. However, overexpression of Ldb16p in the absence of Sei1p causes restoration of TAG levels, suggesting an independent role of Ldb16p in controlling TAG concentration.

## Methods

### Cell strains and cell culture conditions

All yeast strains are based on either BY4742 or the W303-1A haploid cells except when indicated. See Table 1 for strains used. Knock-out or knock-in strains were generated by standard procedures [30]. Drug resistance cassettes conferring resistance to hygromycin B (Hph) and nourseothricin (Nat) [31] were kindly provided by Benjamin Tu (UT Southwestern). SCD-based defined media ([14] with 2 % dextrose) with appropriate supplements for auxotrophies were used for all experiments, except for the experiment shown in Fig. 3, where lipid droplets were induced with galactose [14]. For W303 strain, additional adenine (800 ug/mL) was added to suppress the ade (red) phenotype for fluorescence studies. For log phase and stationary phase, cells were grown for 6 to 8 h, and 16 to 24 h, respectively after back-dilution.

**Table 1:** A list of strains used in this study

Strain Name	Genotype or Description	Ref/Source
BY4741- or 4742-based strains	Mata (BY4741*) or* Mat*α* (BY4742) *his3Δ1 leu2Δ0 lys2Δ0 ura3Δ0*	Open Biosystems
Opi1-GFP	Opi1-GFP::HIS3 (BY4742), a haploid progeny from crossing BY4741 Opi1-GFP::HIS3 and BY4742 *sei1*∆::Kan	Open Biosystems
Scs2-GFP	Scs2-GFP::HIS (BY4742)	Open Biosystems
* sei1Δ*	*sei1Δ::*KanMX (BY4742)	Open Biosystems
* sei1Δ*Opi1-GFP	Opi1-GFP *sei1Δ::*KanMX (BY4742)*,* a haploid progeny from crossing Opi1-GFP::HIS3 (BY4741) and *sei1*∆::Kan (BY4742)	This study
* sei1Δ*Scs2-GFP	Scs2-GFP *sei1Δ::*KanMX (BY4742)*,* a haploid progeny from crossing Scs2-GFP::HIS3 (BY4741) and *sei1*Δ::Kan (BY4742)	This study
Opi1^*FFAT*mut^-GFP	Opi1^*FFATmut*^-GFP::*KanMX6* (BY4742)	This study
* sei1Δ* Opi1^*FFATmut*^-GFP	Opi1^*FFATmut*^-GFP::*KanMX6* (BY4742), a crossed progeny between Opi1^*FFAT*mut^-GFP and *sei1Δ* ::Kan (BY4741)	This study
Pah1-GFP::HIS3	Pah1-GFP::HIS3 (BY4741)	Open Biosystems
* sei1*Δ Pah1-GFP	*sei1*Δ::Hph Pah1-GFP::HIS3 (BY4741)	This study
W303-based strains	*MATa* (W303-1A) *or* MAT*α* (W303-1B)*, leu2-3,112 trp1-1 can1-100 ura3- 1 ade2-1 his3-11,15*	Thomas 1989 [35], Open Biosystems
Opi1-mCherry	Opi1-mCherry::KanMX (W303-1A or 1B)	This study
* sei1Δ*	*sei1Δ::*Hph (W303-1B)	This study
* sei1Δ* Opi1-mCherry	*sei1*Δ::Hph Opi1-mCherry (W303-1B)	This study
Pah1-3xFlag	Pah1-3xFlag::KanMX (W303-1A)	This study
* sei1Δ* Pah-3xFlag	*sei1Δ*::Hph Pah1-3xFlag::KanMX (W303-1A)	This study
* 3KO(*_*Gal*_ *DGA1)* Opi1-mCherry	*3KO(*_*Gal*_ *DGA1)* Opi1-mCherry::KanMX	Cartwright et al., 2015 [14]; this study
* 3KO(*_*Gal*_ *DGA1) sei1Δ* Opi1-mCherry	*3KO(*_*Gal*_ *DGA1) sei1Δ* Opi1-mCherry::KanMX	Cartwright et al., 2015 [14]; this study
* 3KO(*_*Gal*_ *ARE1)* Opi1-mCherry	*3KO(*_*Gal*_ *ARE1)* Opi1-mCherry::KanMX	Cartwright et al. 2015 [14]; this study
* 3KO(*_*Gal*_ *ARE1) sei1Δ* Opi1-mCherry	*3KO(*_*Gal*_ *ARE1) sei1Δ* Opi1-mCherry::KanMX	Cartwright et al., 2015 [14]; this study
* gpt2Δ* Opi1-mCherry	*gpt2Δ*::NAT Opi1-mCherry ::KanMX	This study
* gpt2Δ sei1Δ* Opi1-mCherry	*gpt2Δ*::NAT *sei1*Δ::Hph Opi1-mCherry ::KanMX	This study
* slc1Δ* Opi1-mCherry	*slc1Δ*::NAT Opi1-mCherry ::KanMX	This study
* slc1Δ sei1Δ* Opi1-mCherry	*slc1Δ*::NAT sei1*Δ*::Hph Opi1-mCherry ::KanMX	This study
* ale1Δ* Opi1-mCherry	*ale1Δ*::NAT Opi1-mCherry ::KanMX	This study
* ale1Δ sei1Δ* Opi1-mCherry	*ale1Δ*::NAT *sei1Δ*::Hph Opi1-mCherry ::KanMX	This study
* loa1Δ* Opi1-mCherry	*loa1Δ*::NAT Opi1-mCherry ::KanMX	This study
* loa1Δ sei1Δ* Opi1-mCherry	*loa1Δ*::NAT sei1::Hph Opi1-mCherry ::KanMX	This study
* dgk1Δ* Opi1-mCherry	*dgk1Δ*::NAT Opi1-mCherry ::KanMX	This study
* dgk1Δ sei1Δ* Opi1-mCherry	*dgk1Δ*::NAT *sei1*Δ::Hph *opi1*::Opi1-mCherry KAN	This study
* spo14Δ* Opi1-mCherry	*spo14Δ*::NAT Opi1-mCherry ::KanMX	This study
* spo14Δ sei1Δ* Opi1-mCherry	*spo14Δ*::NAT *sei1*Δ::Hph Opi1-mCherry ::KanMX	This study
* ldb16Δ*	*ldb16Δ*::Nat	This study
* sei1Δ ldb16Δ* (”ΔΔ”)	*sei1Δ*::Hph *ldb*16Δ::Nat	This study
* ldb16∆* Opi1-mCherry	*ldb*16Δ::Nat Opi1-mCherry::Kan	This study
* sei1∆ldb16∆Opi1-mCherry*	*sei1Δ*::Hph *ldb*16Δ::Nat Opi1-mCherry::Kan	This study

### Reagents

Nourseothricin was purchased from Werner Bioagents (Jena Germany). Hygromycin B was purchased from Gold Biotechnology (Goldbio.com, St. Louis, MO). G418 Sulfate (used to select for resistance to the Kan^R^ gene) was purchased from AG Scientific (San Diego, CA).

### Plasmids

Plasmid GFP-Spo20 (51–91) was a gift of Aaron Neiman (Stony Brook University). Plasmids encoding Pah1-GFP and Pah1-D398A/D400A-GFP were kindly provided by Symeon Siniossoglou (University of Cambridge). A TAP tag used in this study was derived form the sequence of HBH TAP tag followed by the KanMX6 marker from plasmid DQ407918 (PK440; pFA6a-HBH-KanMX6 [32], a gift from Anne Spang (Biozentrum, Basel) such that the HBH TAP tag was placed at the carboxy end of the modified protein. The pFA6a mCherry-KanMX6 plasmid was a gift of Chuck Cole (Dartmouth U., Dartmouth, NH). Other plasmids used in this study were summarized in Table 2.

**Table 2:** A list of plasmids used in this study

Plasmids	Description	Ref/source
pRS426 GFP-Spo20(51–91)	pRS426 expressing GFP fused to Spo20(51–91) which is sensing PA	Nakanishi et al., 2004 [23]
YCpLac111-Pah1-GFP	PAH1-GFP under control of the PAH1 promoter into CEN/LEU2 vector	Karanasios et al., 2010 [25]
YCpLac111-Pah1-D398AD400A-GFP	PAH1[D398A D400A]-GFP under control of the PAH1 promoter into CEN/LEU2 vector	Karanasios et al., 2010 [2])
pRS315 PGK1	Yeast centromeric expressing plasmid (YCp) with *LEU2* marker carrying PGK1 promoter/ terminator	Binns et al., 2006 [36]
pRS315 PGK1-CFP-HDEL	pRS315 expressing ER luminal CFP marker	Szymanski et al., 2007 [12]
pRS315 PGK1-Pah1	pRS315 expressing Pah1 under control of *PGK1* promoter/terminator	Adeyo et al., 2011 [37], This study
pRS 315 PGK1-Cds1	pRS315 expressing Cds1 under control of *PGK1* promoter/terminator	This study
pRS 315 PGK1-Dgk1	pRS 315 expressing Dgk1 under control of *PGK1* promoter/terminator	This study
pRS315 PGK1-mCherry	pRS315 expressing mCherry under control of *PGK1* promoter/terminator	Cartwright et al., 2015 [14]
pRS315 PGK1-Sei1-mCherry	pRS315 expressing Sei1-mCherry under control of *PGK1* promoter/terminator	Cartwright et al., 2015 [14]
pRS-315 Sei1^ΔNterm^-mCherry	pRS315 expressing Sei1^ΔNterm^-mCherry under control of *PGK1* promoter/terminator	Cartwright et al., 2015 [14]
pRS315 PGK1-Ldb16	pRS315 expressing Ldb16 under control of *PGK1* promoter/terminator	This study
pRS315 PGK1-Sei1	pRS315 expressing Sei1 under control of *PGK1* promoter/terminator	Szymanski et al., 2007 [12]
pRS315-BSCL2	pRS315 expressing BSCL2 (short isoform) under control of *PGK1* promoter/terminator	Szymanski et al., 2007 [12]
pRS316 PGK1-Sei1-TAP	pRS316 expressing SEI1-TAP fusion protein under control of PGK1 promter/terminator. TAP sequence was amplified from HBH TAP tag (HBH-KanMX6), a gift from Anne Spang (Biozentrum,Basel)	Tagwerker et al., 2006, This study
pRS316 PGK1-Sei1^ΔNterm^ -TAP	pRS316 expressing SEI1^ΔNterm^ -TAP fusion protein under control of PGK1 promter/terminator.	This study
pRS316 PGK1-Ldb16-Sei1-TAP	pRS316 expressing Ldb16-Sei1-TAP fusion protein under control of PGK1 promter/terminator. TAP seq was generated similar way to the above.	This study
pRS316 PGK1-Ldb16-Sei1^ΔNterm^ -TAP	pRS316 expressing Ldb16-Sei1^ΔNterm^ -TAP fusion protein under control of PGK1 promter/terminator. TAP seq was generated similar way to the above.	This study

### Immunoblot

For examining protein expression, whole lysates were prepared in SUME BUFFER (1 % SDS, 8 M Urea, 10 mM MOPS, pH 6.8, 10 mM EDTA, 0.01 % bromophenol blue) from yeast as described [33] and subjected to SDS-PAGE followed by immunoblot. mCherry and FLAG epitope were probed with rabbit polyclonal anti-DsRed (Clontech, 632496) and mouse monoclonal anti-FLAG M2 (Sigma-Aldrich, F3165) antibody, respectively. Rabbit Anti-glucose-6-phosphate dehydrogenase (Zwf1p) antibody (Sigma, A9521) was used for a loading control. Antibodies were detected using infrared-dye conjugated secondary antibodies and LICOR Odyssey detection system.

### Fluorescence microscopy

All microscopy except for the FRAP experiment (Fig. 2d and e) was performed on a Zeiss Axioplan 2E microscope and images were acquired into Slidebook 4.2 or 5.0 (Intelligent Imaging Innovations, Denver, CO) with a SensiCam digital camera (Cooke), as previously described [14]. All images represent single optical planes (approximately halfway through the cell) except for those shown in Fig. 5, which are maximal projection images from a z-stack of ~15 planes spaced 0.3 μ apart. For lipids droplet staining, BODIPY 493/503 was used. To fix the yeast cells, 2 % formaldehyde was used as described before [34].

### FRAP experiment

FRAP experiments were performed on a laser scanning confocal spectral microscope (Zeiss LSM510 Meta) using a 63x oil immersion oil (NA1.4) at room temperature. Cells were put on 2.5 % agarose pads in SC-leu media covered on glass slide as described (ref). GFP fluorescence was detected using the 488 laser line of an Argon laser in conjunction with a BP 500–530 filter.

A defined region of interest (ROI, 0.9 μm in diameter) was photobleached at full laser power at 488 nm (100 % power, 25 iterations) and recovery of fluorescence was monitored by scanning the ROI at low laser power (5 % of laser power) by 10-s interval scans under 4x zoom. Fluorescence intensity was normalized to the prebleach intensity. Images intensity were measured using Image J (v.1.41, NIH).

### Lipid analysis

Lipid extraction and one-dimensional thin layer chromatography were performed as previously described [14]. Lipid bands were digitally scanned and their intensities were determined by ImageJ. Concentrations were calculated relative to serial dilutions of lipid standards.

### Statistical analyses

Prism v. 6 (GraphPad, La Jolla, CA) was used for all statistical analysis. For experiments with multiple groups, one-way ANOVA and the Tukey test were used to determine the degree of significance between samples.

## Availability of supporting data

Additional file 1: Figures S1 and S2 accompany the manuscript.

Additional file 1: Figure S1: Scs2p-GFP can form strong puncta in *sei1Δ* cells, demonstrates puncta of this fluorescent hybrid especially in stationary phase.

Additional file 1: Figure S2: Suppressing supersized droplets with inositol does not inhibit PA puncta formation, using Opi1-mCherry to demonstrate PA puncta.

All supporting data can be found in the manuscript text and its additional files.

## Additional file

Additional file 1:
**Figure S1.** Scs2p-GFP can form strong puncta in *sei1*Δ cells. Shown are GFP fluorescence images in log or stationary phase cells. Scale bar, 5 μm. **Figure S2**. Suppressing supersized droplets with inositol does not inhibit PA puncta formation. (**A**) WT or (**B**) *sei1*Δ cells (both expressing Opi1-mCherry) were grown in SCD ± 75 μM inositol for 6 h. The added inositol suppressed supersized droplets in *sei1*∆ but not PA puncta. Scale bars, 5 μm. (PDF 2388 kb)

## Abbreviations

DAG: diacylglycerol
G-3-P: glycerol-3-phosphate
LPA: lysophosphatidic acid
PA: phosphatidic acid
SE: steryl ester
TAG: triacylglycerol
FRAP: Fluorescent recovery after photobleaching
