# Supplementary material for: Dissecting seipin function: the localized accumulation of phosphatidic acid at ER/LD junctions in the absence of seipin is suppressed by Sei1pΔNterm only in combination with Ldb16p
Source: BMC Cell Biol. 2015 Dec 4;16:29. doi: 10.1186/s12860-015-0075-3 (PMC4670494; doi:10.1186/s12860-015-0075-3)
Supplement: Additional file 1: — Figure S1. Scs2p-GFP can form strong puncta in sei1Δ cells. Shown are GFP fluorescence images in log or stationary phase cells. Scale bar, 5 μm. Figure S2. Suppressing supersized droplets with inositol does not inhibit PA puncta formation. (A) WT or (B) sei1Δ cells (both expressing Opi1-mCherry) were grown in SCD ± 75 μM inositol for 6 h. The added inositol suppressed supersized droplets in sei1∆ but not PA puncta. Scale bars, 5 μm. (PDF 2388 kb) [file 12860_2015_75_MOESM1_ESM.pdf]

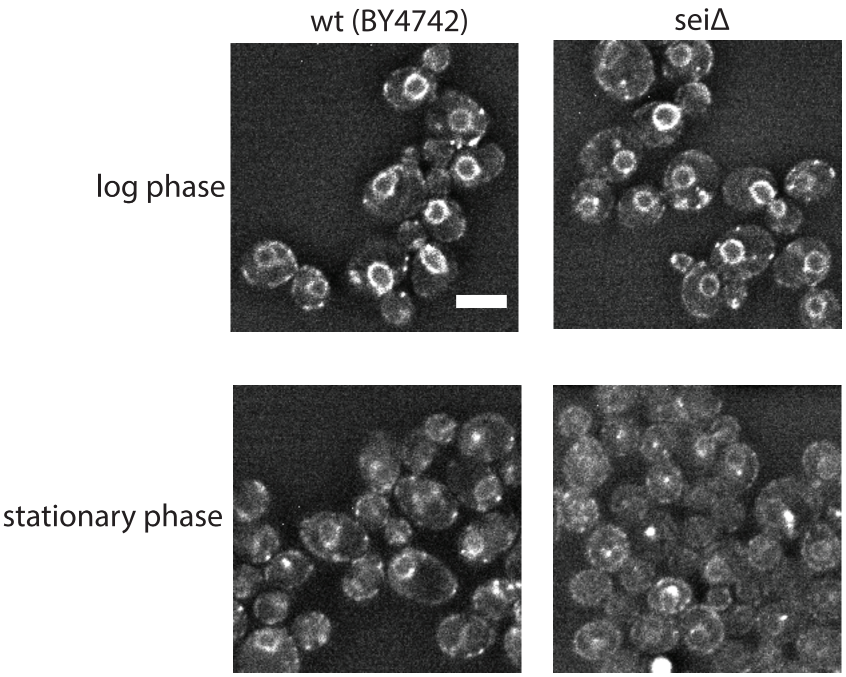

Supplemental Figure S1. Scs2p-GFP can form strong puncta in *sei1*Δ cells. Shown are GFP fluorescence images in log or stationary phase cells. Scale bar, 5 µm.

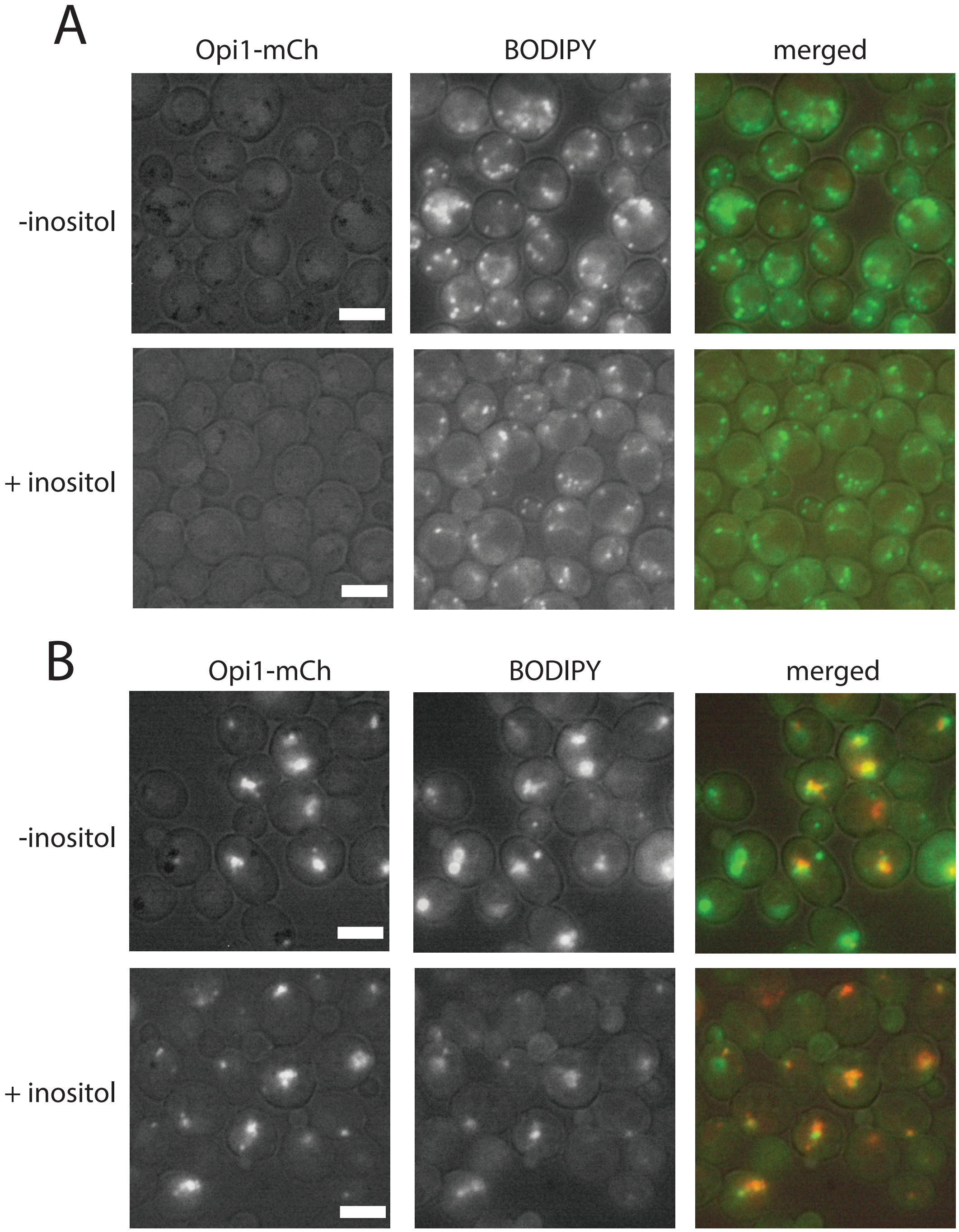

Supplemental Figure S2. Suppressing supersized droplets with inositol does not inhibit PA puncta formation. (A) WT or **(B)** *sei1*Δ cells (both expressing Opi1-mCherry) were grown in SCD ± 75 μM inositol for 6 h. The added inositol suppressed supersized droplets in *sei1*Δ but not PA puncta. Scale bars, 5 μm.
